# Moderating Role of Condom-Use Inertia on the Association Between Status Quo Bias and Pre-Exposure Prophylaxis Resistance Intention Among Chinese Men Who Have Sex With Men: Cross-Sectional Study

**DOI:** 10.2196/88806

**Published:** 2026-04-10

**Authors:** Min Zhao, Shanshan Li, Xiang Zhao, Jincheng Hu, Zhuoheng Yin, Fanpu Ji, Weiming Tang, Lei Zhang

**Affiliations:** 1 Department of Hepatology, Second Affiliated Hospital of Xi'an Jiaotong University, No. 157 Xiwu Rd, Xi'an, Shaanxi, China, 86 15910593477; 2 China-Australia Joint Research Centre for Infectious Diseases, School of Public Health, Xi'an Jiaotong University, Xi'an, Shaanxi, China; 3 Second Department of Coronary Heart Disease, First Affiliated Hospital of Xinjiang Medical University, Urumqi, Xinjiang, China; 4 University of North Carolina at Chapel Hill Project-China, Guangzhou, Guang dong, China; 5 Institute of Global Health and Infectious Diseases, University of North Carolina at Chapel Hill, Chapel Hill, NC, United States; 6 Melbourne Sexual Health Centre, Alfred Health, Melbourne, Victoria, Australia; 7 School of Translational Medicine, Faculty of Medicine, Monash University, Melbourne, Victoria, Australia

**Keywords:** HIV, pre-exposure prophylaxis, men who have sex with men, social norms, condoms, cross-sectional studies, status quo bias, China

## Abstract

**Background:**

While prior studies have examined structural and individual-level barriers to pre-exposure prophylaxis (PrEP) uptake, little is known about the psychological mechanisms underlying resistance to PrEP, particularly among high-risk groups. The status quo bias (SQB) theory provides a theoretical framework for understanding why individuals may resist beneficial health innovations.

**Objective:**

The aim of this study was to examine the associations between SQB and PrEP resistance intention among Chinese men who have sex with men (MSM) and to test whether condom-use inertia moderated these relationships.

**Methods:**

We conducted a cross-sectional online survey among 1022 MSM in China from November 2024 to February 2025. Theory-guided multi-item measures were constructed to capture key dimensions of SQB. Their internal consistency and construct validity were examined using confirmatory factor analysis prior to regression modeling. Multiple linear regression models assessed main effects and moderation effects, adjusting for sociodemographic and behavioral covariates.

**Results:**

A total of 1022 MSM were included in the final analysis (mean age 29.6 y); the majority identified as homosexual (767/1022, 75.1%) and were unmarried (896/1022, 87.7%). Regression analyses revealed that transition costs were positively associated with PrEP resistance intention (*β*=0.26, 95% CI 0.17-0.35; *P*<.001), while social norms were negatively associated (*β*=–0.19, 95% CI –0.27 to –0.12; *P*<.001). Condom-use inertia significantly moderated both associations, amplifying the positive relationship between transition costs and resistance (*β*=0.04, 95% CI 0.01-0.09; *P*=.03) and enhancing the negative association of social norms (*β*=–0.05, 95% CI –0.09 to 0.00; *P*=.04). Subgroup analyses showed that the amplifying effect of condom-use inertia on transition costs was particularly evident among participants with postgraduate education (*β*=0.13, 95% CI 0.01-0.26; *P*=.04). Conversely, its strengthening effect on social norms was more pronounced among MSM 30 years or younger (*β*=–0.11, 95% CI –0.17 to –0.04; *P*=.001) and those identifying as homosexual (*β*=–0.07, 95% CI –0.12 to –0.02; *P*=.01).

**Conclusions:**

This study suggests that SQB is an important psychological barrier to PrEP adoption among Chinese MSM. The moderating role of condom-use inertia highlights the relevance of habitual condom-use routines in shaping PrEP resistance. Behaviorally informed strategies that reduce transition costs and leverage social norms may help mitigate resistance and improve PrEP uptake.

## Introduction

According to the latest statistics from the World Health Organization, an estimated 40.8 million people were living with HIV at the end of 2024, and approximately 1.3 million people acquired HIV in the same year [1]. Men who have sex with men (MSM) continue to be among the populations at highest risk for HIV infection, with a relative risk estimated to be 23 times higher than that of the general population [2,3]. In the ongoing global public health effort to end the HIV epidemic, pre-exposure prophylaxis (PrEP) has emerged as a milestone biomedical intervention. Evidence shows that when taken as prescribed, PrEP can reduce the risk of HIV acquisition among MSM by approximately 99% [4]. Nevertheless, despite its enormous potential to reshape the trajectory of the HIV epidemic, the implementation and scale-up of PrEP continue to encounter considerable challenges.

Implementing and promoting PrEP uptake among MSM remains a considerable challenge. Although studies estimate that over 66.7% of MSM in China express willingness to use PrEP, the actual uptake rate remains below 10% [5,6]. Existing research has focused on 2 broad categories of barriers to PrEP willingness or uptake. On the structural level, a large body of literature highlights obstacles such as limited health care access, high out-of-pocket costs, and service delivery gaps [7,8]. On the psychosocial level, researchers have emphasized the role of factors such as stigma, perceived HIV risk, and self-efficacy [9-11]. While crucial metrics like PrEP willingness and uptake measure the presence of a positive inclination or a final behavior, they may not fully capture the active psychological processes underlying the rejection of PrEP. This specific failure to adopt PrEP among those who need it most underscores the limitations of “willingness” as a construct and necessitates a new focus on the cognitive and behavioral factors driving active resistance intention.

From the perspective of status quo bias (SQB) theory, individuals often prefer preserving their current circumstances, even when alternative options offer greater benefits [12]. This study focuses on 2 principal mechanisms, drawn from SQB theory, that may explain this phenomenon: rational decision-making and psychological commitments [13]. Rational decision-making implies that individuals conduct a cost-benefit analysis before transitioning to a new HIV prevention strategy [13]. Transition costs refer to the burdens associated with adopting a new practice; when such costs outweigh the perceived benefits, individuals are likely to maintain the status quo [14]. In the context of PrEP, these transition costs may present a significant barrier to adoption, including the time and financial investments required for behavioral adjustments, such as medical consultations, regular HIV testing, and the establishment of consistent medication routines [15-17]. For example, Katz et al [18] reported that high-risk women in Kenya experienced difficulties initiating PrEP due to professional scheduling conflicts, educational disruptions, and prohibitive transportation expenses.

Psychological commitment reflects prevailing social norms [19]. In HIV prevention, social norms refer to the collective expectations and shared attitudes within MSM communities regarding PrEP usage. Previous studies suggest that the willingness of MSM to use PrEP is significantly influenced by perceived social approval or disapproval from significant others [20,21]. Specifically, the attitudes and opinions of family members, peers, and healthcare providers can influence the perceptions and decisions of MSM regarding PrEP [22,23]. When individuals are embedded in social networks that actively endorse or discuss PrEP use, these positive normative cues can help counteract status quo tendencies and encourage PrEP uptake [24,25].

Rather than arising from reflective cost-benefit analyses, inertia reflects an automatic reliance on established behavioral patterns and a natural resistance to change [26,27]. While condoms remain a cornerstone of HIV prevention, their effectiveness is often compromised by inconsistent use—a well-documented risk factor, particularly among high-risk MSM [28]. PrEP is a critical supplement to HIV prevention for this high-risk group. However, adopting this biomedical paradigm triggers cognitive resistance. For example, Han et al [29] found that individuals who preferred and consistently used condoms as a method of HIV prevention were significantly less willing to consider PrEP adoption. Similarly, previous research has shown that MSM with high self-efficacy regarding condom use were less likely to contemplate using PrEP [11]. Building on these insights, while recognizing the underexplored relationship between SQB and behavioral inertia, we hypothesize that condom-use inertia moderates this interplay, such that the influence of factors like transition costs and social norms becomes more pronounced among individuals with higher levels of inertia.

Although a growing body of research has explored barriers to PrEP adoption and willingness, few studies have explicitly examined PrEP resistance intention through a behavioral economics lens. Moreover, the potential influence of condom-use inertia has received little empirical attention. Guided by SQB theory, this study examines how transition costs and perceived social norms are associated with PrEP resistance intention among Chinese MSM and tests whether condom-use inertia conditions these associations.

## Methods

### Study Design and Participant Recruitment

We conducted this cross-sectional study using an anonymous, web-based questionnaire deployed via Sojump, a widely used online survey platform in China, and reported the online survey in accordance with the CHERRIES (Checklist for Reporting Results of Internet E-Surveys) (Checklist 1). We collected data from November 29, 2024, to February 11, 2025. Participants were recruited through convenience and snowball sampling. The research team developed recruitment materials, including digital posters and standardized outreach messages, which were disseminated through the WeChat official accounts of collaborating community-based organizations (CBOs) and local Centers for Disease Control and Prevention. Guangdong served as the initial dissemination hub because the core CBO partner responsible for launching recruitment was based there. To implement snowball sampling, participants were encouraged to share the survey link with their peers. As a result, recruitment extended beyond the initiating hub and ultimately reached multiple regions across mainland China, including Guangdong, Hubei, Shandong, Jiangsu, Sichuan, and Shanghai.

Eligible participants were those who met the following inclusion criteria: aged 18 years or older, identified as cisgender male, had a history of sexual activity with other men, were self-reported HIV-negative or unaware of their HIV status, and provided electronic informed consent before beginning the survey. We excluded participants who declined to consent or were deemed unable to complete the survey due to severe psychiatric illness or significant cognitive impairment, as determined by self-report or investigator assessment. We restricted participation to cisgender MSM to preserve conceptual and measurement consistency because the study constructs and items were developed to capture PrEP-related resistance and condom-use routines in this population and have not been validated for transgender and gender-diverse participants.

Before accessing the questionnaire, potential participants viewed an electronic informed consent page that described the study investigator, the study’s purpose, and the estimated survey duration (about 15 min). The consent page also explained what information would be collected, that responses would be stored on a secure, password-protected server managed by the study team, that only authorized investigators would have access, and that the data would be used solely for scientific research. Participants could proceed to the survey only after actively indicating consent (eg, selecting an “I agree” option). Participants whose responses passed the logic-consistency and attention-check procedures received 30 yuan (about US $5) as a token of appreciation for their time.

To ensure data quality, the questionnaire incorporated embedded attention-check items and logic-consistency checks. Of 1311 submitted responses, 257 failed quality control and were excluded, leaving 1054 responses. We then removed 32 duplicate entries identified using the phone numbers submitted with the questionnaire, resulting in 1022 unique participants included in the final analysis (Multimedia Appendix 1). Phone numbers were collected solely for duplicate checking and were removed from the analytic dataset after screening. The survey platform also required completion of mandatory items before submission, minimizing missing data.

The present study focuses on PrEP resistance intention and incorporates multiple covariates and interaction testing. An a priori power analysis using G*Power 3.1.9.7 indicated that, for a multiple linear regression model with *α*=.05 and up to 20 predictors, a sample of 157 participants would be required to detect a medium effect (f²=0.15) with 80% power, whereas 1064 participants would be required to detect a small effect (f²=0.02). The final analytic sample of 1022 participants fell between these 2 benchmarks, suggesting adequate power to detect effects in the small-to-medium range in the primary regression models. However, smaller interaction effects and exploratory subgroup analyses should be interpreted cautiously.

### Ethical Considerations

We obtained ethical approval for this study from the Institutional Review Board of Shenzhen University (protocol PN-202400070). All participants provided informed consent electronically before accessing the survey. The survey was administered anonymously online. Phone numbers were collected only for duplicate screening and compensation purposes and were removed from the analytic dataset after screening. All data were stored on a password-protected server accessible only to the research team and were used solely for research purposes. Participants whose responses passed the quality-control procedures received 30 Yuan (about US $5) as compensation.

### Measures

#### Study Constructs and Measurement

We operationalized the key study constructs using theory-guided, multi-item measures informed by the behavioral economics framework of SQB [13] and by prior empirical literature on PrEP adoption barriers among MSM [15-17,21-25,30,31]. We adapted all items to suit the cultural and behavioral context of the target population and refined them through expert consultation and pilot testing [32]. Participants rated all items on a 7-point Likert scale, ranging from 1 (strongly disagree) to 7 (strongly agree). We then calculated the final score for each dimension as the arithmetic mean of its corresponding items. Table 1 summarizes the study constructs and their items. These constructs were analyzed as distinct measures (outcome, predictors, and moderator) rather than as subscales of a single instrument.

**Table 1.** Study constructs and items.

Study constructs	Items	
Transaction costs		
TC1	It would take a lot of time and effort for PrEP^a^-related physical exams.	
TC2	I would spend a lot of money if I were to switch to using PrEP.	
TC3	There would be health-related side effects if I were to switch to using PrEP.	
TC4	Switching to using PrEP could result in unexpected hassles.	
Social norms		
SN1	My doctors/community outreach workers think I should use PrEP. [reverse-coded item]	
SN2	My family/friends think I should use PrEP. [reverse-coded item]	
SN3	My sex partners think I should use PrEP. [reverse-coded item]	
Condom-use inertia		
I will continue using condoms for preventing HIV…		
CI1	…simply because it is what I have always done.	
CI2	…I enjoy doing so.	
CI3	…even though I know it is not the best way of doing things.	
Resistance intention		
RI1	I oppose the idea of using PrEP as a strategy to prevent HIV.	
RI2	I will not start using PrEP for preventing HIV.	
RI3	I will not comply with the medical instructions to use PrEP for preventing HIV.	

a PrEP: pre-exposure prophylaxis.

#### Primary Outcome

The primary outcome variable, PrEP resistance intention, was assessed using 3 items designed to capture individuals’ intentions to resist the adoption and use of PrEP. These items reflect a spectrum of resistance, ranging from general attitudinal opposition to the change to specific behavioral intentions to reject both the adoption process and the medication regimen. We calculated the outcome score as the mean of the 3 items, with higher scores indicating stronger resistance intentions.

#### Explanatory Variables

We focused on 2 theoretically salient dimensions of SQB as the main explanatory variables: transition costs and perceived social norms. These dimensions reflect distinct psychological mechanisms that may contribute to resistance to behavioral change. Transition costs included 4 items covering concerns about time, financial burden, side effects, and potential hassles associated with PrEP uptake [15-17]. We assessed social norms using 3 reverse-coded items that evaluated perceived approval of PrEP use from key referents, such as health care workers, friends, and sexual partners [21-25]. We reverse-coded these items so that higher values reflected more supportive norms, consistent with the hypothesized protective association.

#### Moderator Variable

Condom-use inertia, reflecting an inertial reliance on behavioral persistence, was measured using 3 items. These items explored the behavioral, affective, and cognitive tendencies of individuals to continue using condoms for HIV prevention, even in cases where condom use might be perceived as suboptimal [9,29,33]. Higher scores indicated more substantial condom-use inertia.

#### Covariates

We selected covariates based on prior empirical evidence identifying their relevance to PrEP-related decision-making and their potential roles as confounding factors [34,35]. Demographic characteristics included age, sexual orientation, marital status, education level, and monthly income. Sexual behavior–related variables included the number of regular male sexual partners and the number of casual male sexual partners reported in the past 6 months. We also included substance use as a behavioral covariate. We asked participants whether they had used any of a list of commonly reported substances within the previous 12 months, specifically in sexual contexts. The checklist included marijuana, cocaine, opium (“white powder”), methamphetamine, poppers, ecstasy, lysergic acid diethylamide, psilocybin, and gamma-hydroxybutyrate or gamma-butyrolactone. We dichotomized responses, coding any substance use during sex as 1 (“yes”) and no reported use as 0 (“no”).

### Statistical Analysis

We used descriptive statistics to summarize participants’ demographic and behavioral characteristics. We reported means and SDs for continuous variables and presented categorical variables as frequencies and proportions. Psychometric evaluation and regression analyses were conducted in the same analytic sample (N=1022). To support the subsequent regression analyses, we evaluated the measurement properties of the theory-guided multi-item constructs. Because the factor structure was specified a priori based on theory, we conducted confirmatory factor analysis (CFA) to evaluate construct validity. We assessed internal consistency using Cronbach α and composite reliability. We examined convergent validity using standardized factor loadings and average variance extracted (AVE) from CFA, and we tested discriminant validity using the Fornell-Larcker criterion by comparing the square root of each construct’s AVE with its correlations with other constructs. We then computed mean scores for all constructs and conducted Pearson correlation analyses to examine the bivariate associations between transition costs, social norms, condom-use inertia, and PrEP resistance intention.

To identify factors associated with PrEP resistance intention, we conducted hierarchical linear regression analyses in 3 steps. Model 1 estimated the main associations between transition costs, social norms, and condom-use inertia and PrEP resistance intention, while adjusting for sociodemographic and behavioral covariates. Model 2 added the transition costs × condom-use inertia interaction, and Model 3 added the social norms × condom-use inertia interaction. We mean-centered continuous predictors before creating interaction terms to reduce multicollinearity and facilitate interpretation. We reported standardized coefficients (*β*), 95% CIs, and *P* values. We assessed model fit using multiple criteria, including *R*²*,* adjusted *R*², and nested model *F* tests, to determine whether interaction terms significantly improved the model fit. We performed all analyses using Stata 17 (StataCorp LLC). Statistical significance was set at *P*<.05 (2-tailed). For exploratory 3-way interaction analyses, *P* values between .05 and .10 were treated as marginal evidence and interpreted cautiously [36,37].

## Results

### Participants’ Sociodemographic Characteristics

The final analytical sample consisted of 1022 Chinese MSM with a mean age of 29.6 (SD 7.6) years. The sample was predominantly homosexual-identified (767/1022, 75.1%), never married (896/1022, 87.7%), and well educated, with 87.8% (897/1022) holding a bachelor’s degree or higher. Regarding monthly income, over one-third (369/1022, 36.1%) earned more than 8000 Yuan (about US $1157) monthly, while 13.3% (136/1022) earned less than 3000 Yuan (about US $434). Participants reported diverse sexual partnership patterns: 57.4% (587/1022) had 1 regular male partner, while 69.8% (714/1022) reported having casual partners in the past 6 months. Nearly one-quarter (253/1022, 24.8%) reported substance use during sexual activities (Table 2).

**Table 2.** Participants’ sociodemographics.

Characteristic	Participants
Age (y)	
Mean (SD)	29.6 (7.6)
≤30, n (%)	658 (64.4)
>30, n (%)	364 (35.6)
Sexual orientation, n (%)	
Homosexual	767 (75.1)
Bisexual	223 (21.8)
Heterosexual	28 (2.7)
Unsure or other	4 (0.4)
Marital status, n (%)	
Never married	896 (87.7)
Engaged or married	78 (7.6)
Separated, divorced, or widowed	48 (4.7)
Highest education level, n (%)	
High school or below	125 (12.2)
Bachelor’s degree	739 (72.3)
Graduate degree or above	158 (15.5)
Monthly income (Yuan)^a^, n (%)	
≤3000	136 (13.3)
3001‐5000	192 (18.8)
5001‐8000	325 (31.8)
>8000	369 (36.1)
Number of regular male partners, n (%)	
0	163 (16.0)
1	587 (57.4)
≥2	272 (26.6)
Number of casual male partners, n (%)	
0	308 (30.2)
1	398 (38.9)
≥2	316 (30.9)
Substance use during sexual activities, n (%)	
No	769 (75.2)
Yes	253 (24.8)

a At the time of analysis, CNY 1≈US $0.145.

### Measurement Properties of Study Constructs

**Figure 1.**
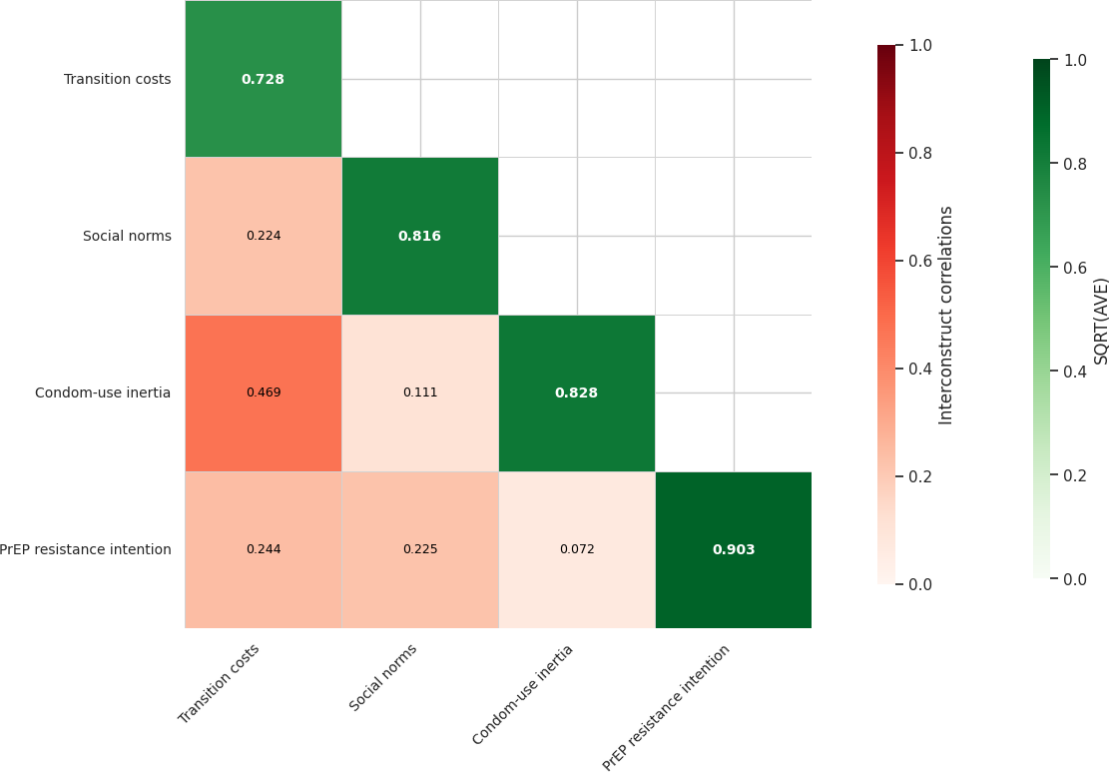
Discriminant validity assessment based on the Fornell-Larcker criterion. AVE: average variance extracted; PrEP: pre-exposure prophylaxis; SQRT: square root.

To support the regression analyses, we assessed the measurement properties of the theory-guided constructs. CFA demonstrated acceptable model fit for the measurement model (*χ*²_79_=523.8, *P*<.001; root mean square error of approximation=0.074, 90% CI 0.068 to 0.08; standardized root mean square residual=0.058; comparative fit index=0.947; Tucker-Lewis index=0.930) (Multimedia Appendix 2). All constructs demonstrated acceptable internal consistency and convergent validity. Internal consistency was excellent for PrEP resistance intention (Cronbach *α*=0.93) and good for condom-use inertia (*α*=.86), social norms (*α*=.85), and transition costs (*α*=.83). Composite reliability values exceeded 0.80 for all constructs. AVE values surpassed the 0.50 threshold, supporting convergent validity (Figure 1).

### SQB Variables by PrEP Resistance Intention

Bivariate correlations revealed consistent associations (Multimedia Appendix 3). Transition costs (mean 4.7, SD 1.1) showed a small-to-medium positive correlation with PrEP resistance intention (mean 3.2, SD 1.6; *r*=0.21; *P*<.001), indicating that higher perceived switching costs to PrEP were associated with greater resistance. Social norms (mean 3.8, SD 1.2) showed a negative correlation of similar magnitude (*r*=–0.21; *P*<.001), suggesting that supportive social environments were associated with lower resistance. Condom-use inertia (mean 4.9, SD 1.3) showed a statistically significant but practically small association with resistance (*r*=0.08; *P*=.008), suggesting that its primary role might be as a moderator rather than a direct predictor.

### Main and Moderation Analysis of PrEP Resistance Intention

Table 3 presents the results of the multiple linear regression models. In model 1, both SQB dimensions were significantly associated with PrEP resistance intention after adjusting for covariates. Transition costs were positively associated with PrEP resistance intention (*β*=0.26, 95% CI 0.17-0.35; *P*<.001). Social norms showed a comparable negative association (*β*=–0.19, 95% CI –0.27 to –0.12; *P*<.001), with more supportive normative environments linked to lower PrEP resistance intention. Condom-use inertia showed no significant direct association (*β*=–0.01, 95% CI –0.08 to 0.07; *P*=.80), supporting its hypothesized role as a moderator rather than a direct predictor of resistance intention.

**Table 3.** Main associations of status quo bias with pre-exposure prophylaxis resistance intentions and the moderating role of condom-use inertia^a^.

Variables	Model 1: *β* (95% CI)^b^	Model 2: *β* (95% CI)^c^	Model 3: *β* (95% CI)^d^
Age (y)	–0.02 (–0.03 to –0)^e^	–0.02 (–0.03 to –0)^e^	–0.02 (–0.03 to –0)^e^
Sexual orientation (ref: Heterosexual)			
Homosexual	–1.57 (–2.14 to –1)^f^	–1.54 (–2.11 to –0.97)^f^	–1.58 (–2.15 to –1.01)^f^
Bisexual	–1.26 (–1.86 to –0.67)^f^	–1.24 (–1.83 to –0.65)^f^	–1.29 (–1.88 to –0.70)^f^
Unsure or other	–1.54 (–3.07 to –0.02)^e^	–1.51 (–3.04 to 0.01)^g^	–1.59 (–3.12 to –0.07)^e^
Marital status (ref: never married)			
Engaged or married	0.14 (–0.23 to 0.51)	0.12 (–0.25 to 0.49)	0.14 (–0.23 to 0.50)
Separated, divorced, or widowed	0.69 (0.22 to 1.15)^h^	0.66 (0.19 to 1.12)^h^	0.68 (0.22 to 1.15)^h^
Highest education level (ref: high school or below)			
Bachelor’s degree	–0.76 (–1.05 to –0.47)^f^	–0.76 (–1.05 to –0.46)^f^	–0.75 (–1.04 to –0.46)^f^
Graduate degree or above	–0.79 (–1.16 to –0.43)^f^	–0.79 (–1.15 to –0.43)^f^	–0.78 (–1.15 to –0.42)^f^
Monthly income (Yuan)^i^ (ref: ≤3000)			
3001‐5000	–0.07 (–0.39 to 0.25)	–0.08 (–0.39 to 0.24)	–0.08 (–0.40 to 0.24)
5001‐8000	0.08 (–0.22 to 0.37)	0.07 (–0.22 to 0.37)	0.07 (–0.22 to 0.37)
>8000	–0.01 (–0.31 to 0.29)	–0.01 (–0.31 to 0.29)	–0.02 (–0.32 to 0.28)
Number of regular male partners (ref: 0)			
1	–0.15 (–0.41 to 0.11)	–0.15 (–0.41 to 0.11)	–0.16 (–0.42 to 0.10)
≥2	0.06 (–0.23 to 0.36)	0.06 (–0.23 to 0.35)	0.06 (–0.23 to 0.35)
Number of casual male partners (ref: 0)			
1	0.31 (0.09 to 0.53)^h^	0.30 (0.08 to 0.52)^h^	0.31 (0.09 to 0.53)^h^
≥2	–0.47 (–0.72 to –0.21)^f^	–0.48 (–0.73 to –0.22)^f^	–0.46 (–0.72 to –0.21)^f^
Substance use (ref: no)			
Yes	–0.17 (–0.38 to 0.04)	–0.18 (–0.39 to 0.04)	–0.16 (–0.37 to 0.05)
Transition costs	0.26 (0.17 to 0.35)^f^	0.26 (0.17 to 0.35)^f^	0.27 (0.18 to 0.35)^f^
Social norms	–0.19 (–0.27 to –0.12)^f^	–0.19 (–0.27 to –0.12)^f^	–0.17 (–0.25 to –0.09)^f^
Inertia	–0.01 (–0.08 to 0.07)	0.00 (–0.07 to 0.08)	–0.00 (–0.08 to 0.08)
Transition costs × inertia	N/A^j^	0.04 (0.01 to 0.09)^e^	N/A
Social norms × inertia	N/A	N/A	–0.05 (–0.09 to –0)^e^

a Coefficients are shown with 95% CIs in parentheses. The *F* test for Δ*R*2 indicates whether the variables added in each step significantly improved the model’s explanatory power compared with the previous model.

b Model 1: Adjusted *R*²=0.234.

c Model 2: Adjusted *R*²=0.247; ΔAdjusted *R*²=0.013; *F* test for Δ*R*²=16.3g.

d Model 3: Adjusted *R*²=0.243; ΔAdjusted *R*²=0.009; *F* test for ΔR²=10.8f.

e *P*<.05.

f *P*<.001.

g *P*<.10.

h *P*<.01.

i At the time of analysis, CNY 1≈US $0.145.

j N/A: not applicable.

Both hypothesized interactions were statistically significant, supporting the moderating role of condom-use inertia in SQB mechanisms. The transition costs × condom-use inertia interaction was significant (*β*=0.04, 95% CI 0.01-0.09; *P*=.03), indicating that the positive association between transition costs and resistance intention was stronger among individuals with higher condom-use inertia. The social norms × condom-use inertia interaction was also significant (*β*=–0.05, 95% CI –0.09 to 0; *P*=.04), indicating that the negative association between supportive social norms and PrEP resistance intention was more pronounced among MSM with high condom-use inertia.

### Moderating Role of Condom-Use Inertia Across Subgroups

Table 4 summarizes the patterns of these moderation effects across various subgroups. The amplifying influence of condom-use inertia on transition costs was particularly pronounced among several subgroups, including participants who were divorced, separated, or widowed (*β*=0.28, 95% CI 0.13-0.43; *P*<.001), those with a postgraduate degree (*β*=0.13, 95% CI 0.01-0.26; *P*=.04), individuals with 1 regular male partner (*β*=0.08, 95% CI 0.01-0.15; *P*=.02), and participants reporting no substance use (*β*=0.07, 95% CI 0.01-0.12; *P*=.01).

**Table 4.** Exploratory analysis of 3-way interactions among status quo bias, condom-use inertia, and covariates^a^.

Characteristic	Transition costs × condom inertia	*P* value	Social norms × condom inertia	*P* value
Age (y)				
≤30	0.07 (–0.00 to 0.13)^b^	.05	–0.11 (–0.17 to –0.04)^c^	.001
>30	0.03 (–0.03 to 0.09)	.37	0.02 (–0.05 to 0.08)	.61
Sexual orientation				
Homosexual	0.04 (–0.02 to 0.09)	.21	–0.07 (–0.12 to –0.02)^d^	.01
Bisexual	0.05 (–0.03 to 0.14)	.23	0.01 (–0.08 to 0.10)	.85
Heterosexual	0.18 (–0.17 to 0.54)	.32	0.05 (–0.23 to 0.33)	.72
Unsure or other	–1.69 (–4.85 to 1.47)	.29	0.12 (–1.08 to 1.32)	.85
Marital status				
Never married	0.04 (–0.01 to 0.10)	.13	–0.06 (–0.11 to –0.01)^d^	.01
Engaged or married	–0.05 (–0.16 to 0.06)	.35	0.06 (–0.06 to 0.18)	.33
Separated, divorced, or widowed	0.28 (0.13 to 0.43)^c^	<.001	–0.08 (–0.26 to 0.11)	.42
Highest education level				
High school or below	0.01 (–0.10 to 0.12)	.84	0.02 (–0.10 to 0.14)	.73
Bachelor’s degree	0.04 (–0.02 to 0.09)	.21	–0.06 (–0.11 to –0)^d^	.04
Graduate degree or above	0.13 (0.01 to 0.26)^d^	.04	–0.07 (–0.18 to 0.03)	.18
Monthly income (Yuan)				
≤5000	0.07 (–0.01 to 0.15)	.10	–0.17 (–0.38 to 0.04)	.11
>5000	0.03 (–0.02 to 0.09)	.24	–0.05 (–0.13 to 0.03)	.21
Number of regular male partners				
0	0.03 (–0.07 to 0.13)	.54	–0.01 (–0.10 to 0.08)	.76
1	0.08 (0.01 to 0.15)^d^	.02	–0.03 (–0.10 to 0.04)	.35
≥2	0.01 (–0.07 to 0.09)	.86	–0.10 (–0.18 to –0.02)^d^	.01
Number of casual male partners				
0	0.09 (–0.02 to 0.19)	.10	–0.12 (–0.23 to –0.02)^d^	.03
1	0.02 (–0.04 to 0.08)	.50	0 (–0.06 to 0.06)	.96
≥2	0.06 (–0.02 to 0.15)	.15	–0.09 (–0.17 to –0.01)^d^	.03
Substance use				
No	0.07 (0.01 to 0.12)^d^	.01	–0.04 (–0.09 to 0.02)	.18
Yes	–0.02 (–0.11 to 0.06)	.60	–0.07 (–0.15 to 0.01)^b^	.09

a The results are shown after controlling for all covariates and status quo bias variables.

b *P*<.1*.*

c *P*<.001*.*

d *P*<.05.

Conversely, the enhancing influence of condom-use inertia on the negative association with social norms was most evident among younger MSM (aged ≤30 y: *β*=–0.11, 95% CI –0.17 to –0.04; *P*=.001), homosexual-identified men (*β*=–0.07, 95% CI –0.12 to –0.02; *P*=.01), unmarried individuals (*β*=–0.06, 95% CI –0.11 to –0.01; *P*=.01), those with a bachelor’s degree (*β*=–0.06, 95% CI –0.11 to 0; *P*=.04), individuals with 2 or more regular partners (*β*=–0.10, 95% CI –0.18 to –0.02; *P*=.01), and those with no casual partners (*β*=–0.12, 95% CI –0.23 to –0.02; *P*=.03).

## Discussion

### Principal Findings

This study examines the psychological mechanisms underlying resistance to PrEP adoption among Chinese MSM, focusing on key dimensions of SQB—transition costs and social norms—and the moderating role of condom-use inertia. Our findings show that both dimensions of SQB are significantly associated with PrEP resistance intention, with condom-use inertia playing a notable moderating role across psychological pathways. This study also highlights the heterogeneity of decision-making processes within MSM populations and may help explain why resistance to PrEP adoption persists despite increasing accessibility. These insights move beyond identifying descriptive barriers to illuminating the specific cognitive and behavioral processes at play, informing the design of behaviorally informed public health strategies.

This study provides empirical evidence on associations between SQB-related factors and PrEP resistance intention among MSM. Our findings suggest that PrEP resistance intention was higher among individuals reporting high transition costs, specifically in terms of time investment, financial costs, and potential side effects. National guidelines recommend HIV testing 1 month after PrEP initiation and quarterly follow-up thereafter, including renal and liver function monitoring [38]. Previous studies indicate that obtaining PrEP through licensed clinics in China is often perceived as time-consuming, requiring multiple visits for testing, prescription, and follow-up [8,39]. Limited provider availability has led some MSM to seek PrEP through informal channels (eg, Dài-Gòu), raising concerns about drug safety [39]. In addition, PrEP in China is largely paid for out of pocket, including medication and required clinical monitoring, and cost has consistently been reported as a major barrier among MSM [40]. Conversely, a supportive social environment was associated with lower resistance, highlighting the inverse relationship with perceived social norms. This pattern aligns with previous literature linking transition costs and normative factors to PrEP-related attitudes and behaviors [24,25,41], and our study extends this work by explicitly framing the outcome as PrEP resistance intention, providing more comprehensive evidence on the active rejection process.

We also identified condom-use inertia as a significant psychological moderator in PrEP decision-making. Although habitual condom use is protective and remains essential for preventing a wide range of sexually transmitted infections, a strong reliance on this established routine may be associated with psychological friction when individuals consider adding or transitioning to PrEP. This friction may relate to the cognitive stability and predictability associated with long-standing habits [42,43]. In this context, condom-use inertia may act as a powerful cognitive filter that reinforces the status quo and amplifies the perceived costs of behavioral change [44,45]. Our findings indicated that men with entrenched condom-use habits perceived the costs and hassles of initiating PrEP as significantly more burdensome, which was associated with greater resistance. Therefore, addressing inertia may help reduce the extent to which established condom-use routines are associated with PrEP resistance.

Paradoxically, this same inertia also amplified the positive association with social norms, suggesting a dual-amplifier mechanism. Individuals with strong habits appear particularly responsive to normative cues, potentially because such cues can penetrate habitual cognitive filters more effectively than informational messages alone [46]. This finding suggests the moderating role of inertia: it strengthens resistance to change when signals support maintaining existing condom routines, yet enhances receptiveness when signals favor PrEP adoption. This dual-amplifier mechanism is particularly relevant for MSM who meet clinical criteria for PrEP, including those with higher sexual activity, multiple recent partners, or increased HIV exposure risk. For these groups, PrEP does not replace condoms but provides supplementary HIV-specific protection where condom effectiveness may be inconsistent or challenged by real-world sexual practices [47].

Exploratory subgroup analyses suggested that the moderating role of condom-use inertia varies across demographic and behavioral profiles. The interaction between condom-use inertia and transition costs was more pronounced among participants with a postgraduate degree, MSM with 1 regular partner, and those reporting no substance use. For example, among participants with higher education, prior research suggests a higher likelihood of reporting consistent condom use [48]. When condom-use inertia is high, PrEP may be perceived as unnecessary and more burdensome to initiate and maintain, magnifying perceived transition burdens and being linked to higher PrEP resistance intention. Along similar lines, MSM reporting one regular partner or no substance use may perceive condoms as sufficiently protective for their current risk profile; high condom-use inertia may therefore make PrEP-related transition costs seem less justified, reinforcing PrEP resistance intention. This suggests that rigid behavioral routines may heighten the psychological burden of initiating PrEP among these groups. Strategies consistent with the MINDSPACE (Messenger, Incentives, Norms, Defaults, Salience, Priming, Affect, Commitments, Ego) framework, such as simplified defaults for PrEP initiation, streamlined refill procedures, and increased salience of concrete PrEP benefits, may help mitigate perceived transition barriers [49,50]. For instance, PrEP eligibility screening or counseling could be embedded within routine HIV and sexual health services using an opt-out default, which may reduce initiation friction. Follow-up and refills could be streamlined (eg, 1-click scheduling, prefilled forms, and automated reminders), which may lower perceived time and hassle costs [51,52].

In contrast, the interaction between condom-use inertia and social norms was most evident among younger MSM, homosexual-identified men, unmarried individuals, those with a bachelor’s degree, and MSM with multiple regular partners, with inertia appearing to strengthen the negative association with social norms. Younger MSM and homosexual-identified men are often more embedded in MSM peer networks and community interactions, where HIV prevention information, attitudes, and behavioral modeling circulate more frequently [53]. Condom-use inertia may reflect the cumulative effects of prior prevention socialization, including repeated reinforcement of condom-related norms, skills, and confidence within peer and sexual networks [54,55]. Within this normative context, individuals anchored in condom-use routines may be more responsive to supportive social endorsement, which may increase the perceived acceptability and feasibility of PrEP and lower resistance intention. Accordingly, norm-based nudges could increase the visibility of supportive norms in MSM social spaces by communicating descriptive and injunctive norms that normalize PrEP consideration. Credible messengers, including peer leaders, PrEP-experienced MSM, and trusted clinicians, may further strengthen normative influence and enhance the perceived feasibility of initiation and persistence [56,57].

### Limitations

Several limitations of this study should be considered when interpreting the results, and these also point to directions for future research. First, this study employed a cross-sectional design, with all variables measured at a single time point. Although this design can identify associations between variables, it does not allow for temporal ordering or causal inference. Second, the sample for this study was drawn from the Chinese cultural and demographic context. While the findings provide valuable localized insights into the PrEP decision-making process among this group, their generalizability may be limited. The factors influencing SQB, such as the nature of social norms and the specific components of transition costs, may differ significantly across other cultural, social, and healthcare system contexts. Third, our reliance on online convenience and snowball sampling limits generalizability to all MSM in China. Recruitment via partner CBO or Centers for Disease Control and Prevention channels and peer sharing likely over-sampled younger, urban, and highly educated individuals, potentially under-representing MSM with lower educational attainment who may differ in HIV prevention knowledge, digital health literacy, and access to PrEP services. Finally, we assessed the prespecified measurement structure using CFA and standard discriminant validity checks; however, we did not conduct exploratory factor analysis or validate the measurement model in an independent sample. Future studies should replicate the factor structure in separate samples and consider incorporating exploratory factor analysis to further evaluate construct dimensionality and strengthen the psychometric evidence base.

### Conclusion

This study applied the theory of SQB to explain PrEP resistance intention, offering a novel theoretical perspective and empirical tools for understanding the intention-behavior gap in public health. Crucially, we identified the moderating role of condom-use inertia, highlighting the cognitive friction involved in shifting from established condom-based routines to new prevention methods. In conclusion, our findings confirm that SQB is an important yet often overlooked factor associated with PrEP adoption among MSM. Integrating insights from behavioral economics into HIV prevention strategies has significant theoretical and practical importance for addressing challenges in PrEP promotion and for informing more effective public health interventions.

## Supplementary material

10.2196/88806 Multimedia Appendix 1 Participant flow diagram.

10.2196/88806 Multimedia Appendix 2 Reliability and convergent validity.

10.2196/88806 Multimedia Appendix 3 Bivariate associations of study constructs with pre-exposure prophylaxis resistance intention.

10.2196/88806 Checklist 1 CHERRIES checklist.

## Abbreviations

AVE: average variance extracted
CBO: community-based organization
CFA: confirmatory factor analysis
CHERRIES: Checklist for Reporting Results of Internet E-Surveys
MINDSPACE: Messenger, Incentives, Norms, Defaults, Salience, Priming, Affect, Commitments, Ego
MSM: men who have sex with men
PrEP: pre-exposure prophylaxis
SQB: status quo bias
