# Supplementary figures and images for: Moderating Role of Condom-Use Inertia on the Association Between Status Quo Bias and Pre-Exposure Prophylaxis Resistance Intention Among Chinese Men Who Have Sex With Men: Cross-Sectional Study
Source: JMIR Public Health Surveill. 2026 Apr 10;12:e88806. doi: 10.2196/88806 (PMC13068365; doi:10.2196/88806)

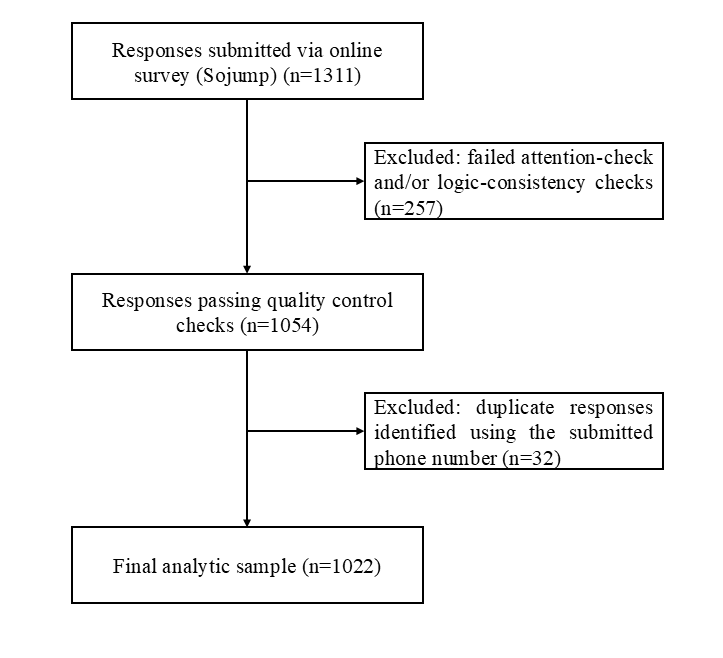

Supplement: Multimedia Appendix 1 [file publichealth-v12-e88806-s001.png]

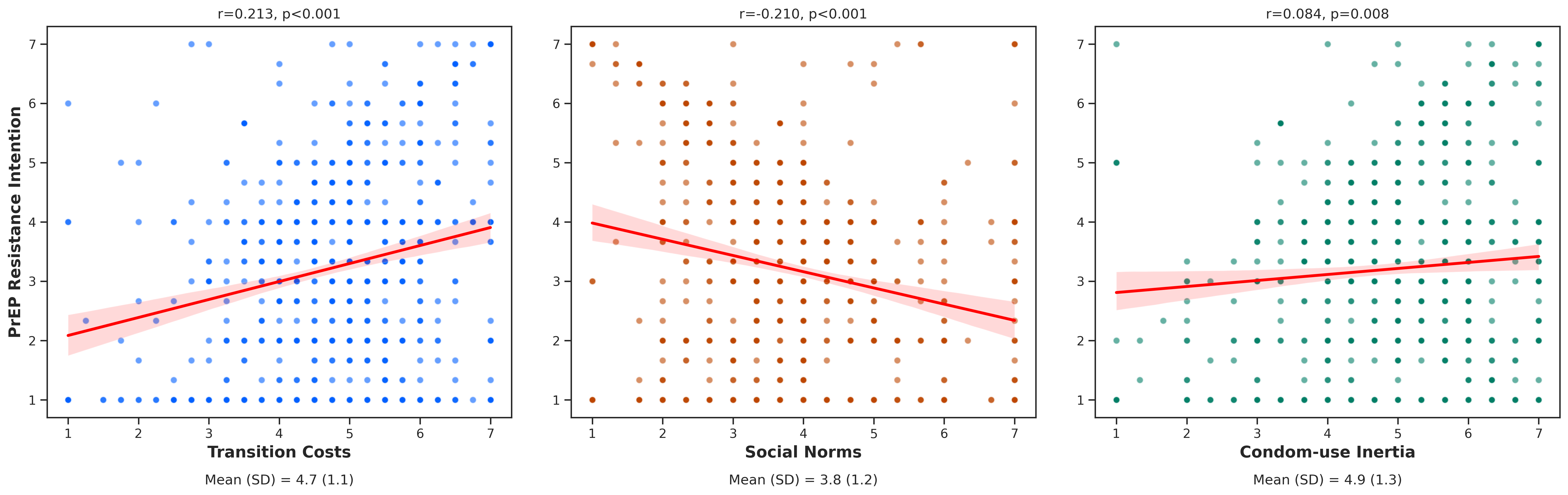

Supplement: Multimedia Appendix 3 [file publichealth-v12-e88806-s003.png]
