# Supplementary material for: Moderating Role of Condom-Use Inertia on the Association Between Status Quo Bias and Pre-Exposure Prophylaxis Resistance Intention Among Chinese Men Who Have Sex With Men: Cross-Sectional Study
Source: JMIR Public Health Surveill. 2026 Apr 10;12:e88806. doi: 10.2196/88806 (PMC13068365; doi:10.2196/88806)
Supplement: Multimedia Appendix 2 [file publichealth-v12-e88806-s002.docx]

Multimedia Appendix 2. Reliability and convergent validity

| **Dimension** | **Cronbach's alpha (α)** | **Composite reliability (CR)** | **Average variance extracted (AVE)** | **Item** | **loading** | **T-statistics** |
| --- | --- | --- | --- | --- | --- | --- |
| Transition Costs | 0.829 | 0.817 | 0.535 | TC1 | 0.704 | 29.39 |
|  |  |  |  | TC2 | 0.685 | 30.92 |
|  |  |  |  | TC3 | 0.781 | 46.82 |
|  |  |  |  | TC4 | 0.750 | 38.51 |
| Social Norms | 0.854 | 0.856 | 0.667 | SN1 | 0.731 | 41.29 |
|  |  |  |  | SN2 | 0.870 | 63.36 |
|  |  |  |  | SN3 | 0.842 | 58.98 |
| Condom-use Inertia | 0.862 | 0.866 | 0.685 | CI1 | 0.894 | 75.13 |
|  |  |  |  | CI2 | 0.851 | 66.18 |
|  |  |  |  | CI3 | 0.729 | 42.05 |
| Resistance Intention | 0.928 | 0.929 | 0.815 | RI1 | 0.827 | 74.82 |
|  |  |  |  | RI2 | 0.945 | 147.67 |
|  |  |  |  | RI3 | 0.932 | 137.97 |

χ²= 523.75, p < 0.001; RMSEA = 0.074, 90% CI (0.068, 0.080); SRMR = 0.058; CFI = 0.947; TLI = 0.930)
